# Adaptive variation in vein placement underpins diversity in a major Neotropical plant radiation

**DOI:** 10.1007/s00442-017-3956-7

**Published:** 2017-09-15

**Authors:** Jamie Males

**Affiliations:** Department of Plant Sciences, University of Cambridge, Cambridge, UK

**Keywords:** Anatomy, Vasculature, Vascular epiphytes, Bromeliaceae, Leaf hydraulics

## Abstract

Vein placement has been hypothesised to control leaf hydraulic properties, but the ecophysiological significance of variation in vein placement in the angiosperms has remained poorly understood. The highly diverse Neotropical Bromeliaceae offers an excellent system for exploring understudied relationships between leaf vein placement, physiological functions, and species ecology. To test key hypotheses regarding the links between vein placement, functional type divergences, and ecological diversity in the Bromeliaceae, I characterised the ratio of interveinal distance (IVD) to vein-epidermis distance (VED) in 376 species, representing all major functional types and 10% of the species diversity in the family, as well as bioclimatic properties and key leaf traits for subsets of species. There were significant differences in vein placement parameters in species of contrasting functional type, habitat association, and bioclimatic distribution. In many C_3_ tank-epiphytes, a greater ratio between interveinal distance and the depth of veins within the mesophyll reflects optimisation for resource foraging in shady, humid habitats. In succulent terrestrials, overinvestment in veins probably facilitates rapid recharge of water storage tissue, as well as restricting water loss. These results highlight how divergences in vein placement relate to distinctive ecophysiological strategies between and within bromeliad functional types, and provide timely insights into how structural–functional innovation has impacted the evolution of ecological diversity in a major radiation of tropical herbaceous angiosperms.

**Electronic supplementary material:**

The online version of this article (doi:10.1007/s00442-017-3956-7) contains supplementary material, which is available to authorized users.

## Introduction

Leaf veins are highly multifunctional structures, but one of their key roles is the transport of xylary water to irrigate the mesophyll and meet transpirational demand (Sack and Scoffoni 2013). The arrangement of veins in the three-dimensional space of the leaf interacts via leaf hydraulic conductance and transpiration with micro-environmental factors to affect the homogeneity of water potential distribution across the lamina. If homogeneous lamina water potential is assumed to promote maximal leaf-level productivity, these structure–function relationships could have important consequences for whole-plant growth and viability (Zwieniecki and Boyce 2014). Experimentation with artificial biomimetic leaves by Noblin et al. (2008) suggested that the optimal arrangement for hydraulic efficiency should be achieved by the equalisation of the interveinal distance (IVD) and the average distance between the primary water-conducting xylem elements in vascular bundles and the stomatiferous epidermis (VED). Zwieniecki and Boyce (2014) demonstrated a shift towards near-equal values of IVD and VED during angiosperm evolution. Alongside other factors such as the origin of xylem vessels, this may have been a key driver of the evolution of increased hydraulic efficiency of the angiosperms and their proliferation into diverse ecological niches (Boyce et al. 2009).

While most angiosperm species examined by Zwieniecki and Boyce (2014) fell close to the IVD = VED line, there were exceptions in the basal monocots, where IVD > VED. The authors described these species as ‘underinvesting’ in veins, which are relatively situated close to the epidermis and widely spaced through the lamina. This type of vein placement was hypothesised by Zwieniecki and Boyce to be physiologically permissible only in humid, shaded environments where heterogeneous epidermal water potential is unlikely to be propagated and impose excessive costs on plant carbon balance. If there exists a developmental link between leaf width and IVD (Dengler and Kang 2001), then high IVD might additionally be associated with the broadening of leaf blades for enhanced light or water interception, while low VED could reduce area-specific leaf construction costs.

Meanwhile, vascular ‘overinvestment’ (IVD < VED) was not observed by Zwieniecki and Boyce (2014) in their species set, and they considered that this hypothetical scenario would generally be physiologically disadvantageous since it would involve the replacement of photosynthetic mesophyll cells with hydraulically redundant vascular bundles. However, vascular overinvestment has more recently been shown by de Boer et al. (2016) to reduce hydraulic limitations on gas exchange in arid-zone Australian eucalypts, suggesting that in specific circumstances this type of vein placement could be advantageous. A different scenario where vascular overinvestment might also be hypothesised to occur is where veins are involved in the rapid recharge of the hydraulic capacitance of succulent tissues during brief pulses of water availability (Griffiths 2013; Ogburn and Edwards 2013). In drought-avoiding succulent xerophytes, a low IVD:VED ratio could also increase the resistance to transpirational water loss by increasing the hydraulic path length in the extra-xylary compartment (Noblin et al. 2008).

The Bromeliaceae (Poales) is an excellent system in which to study the ecological significance of variation in vein placement. This highly diverse family of monocotyledonous angiosperms occurs across the Neotropics and into the North and South American subtropical and temperate zones (Benzing 2000). Bromeliads display a huge variety of morphologies, habits and ecologies, ranging from giant alpine rosettes to diminutive epiphytes. Several schemes for the classification of functional types of bromeliads have been proposed (Pittendrigh 1948; Benzing 2000). A straightforward classification based on photosynthetic pathway, habit, and growth-form could include the following series of functional types: C_3_ mesic terrestrials, C_3_ or Crassulacean acid metabolism (CAM) succulent terrestrials, C_3_ tank-epiphytes, CAM tank-epiphytes, and CAM atmospheric epiphytes. Tank-epiphytes produce a rosette of overlapping leaves that can capture significant quantities of water, leaf-litter and canopy biota, and water and nutrients can be taken up through leaf bases by absorptive foliar trichomes (Benzing 2000). Atmospheric epiphytes (all in the genus *Tillandsia*) are morphologically reduced and display extremely high densities of trichomes, which they use to take up water during brief pulses of availability in highly exposed micro-environments (Reyes-García et al. 2008, 2012). These functional types contain a considerable amount of within-group variation in anatomical and physiological traits, but differences in ecophysiological strategy between functional types are particularly pronounced (Males and Griffiths 2017a). All the morphological and physiological innovations underpinning the functional types have evolved convergently in multiple bromeliad lineages, and this evolutionary replication makes the bromeliads an excellent natural system for researchers interested in the role of structure–function relationships in the generation of ecological diversity (Males 2016). While previous work has highlighted the importance of physiological and anatomical leaf traits in shaping the distinctive properties of bromeliad functional types and their evolutionary diversification (Givnish et al. 2014; Silvestro et al. 2014; Males and Griffiths 2017a), many potentially important aspects of leaf form have received little attention. Among these neglected traits is leaf vein placement. Some bromeliads, notably several ecologically important radiations of C_3_ tank-epiphytes, are restricted to very moist, low-light microclimates (Benzing 2000), and might therefore be good candidates to search for underinvestment-type vein placement among the derived monocots. Meanwhile, limited seasonal water availability is characteristic of the environments in which many xerophytic bromeliads with well-developed succulent water storage tissue occur, making these species contenders for vascular overinvestment.

On the basis of the foregoing considerations, three core hypotheses were formulated regarding the evolution and ecological significance of vein placement in the Bromeliaceae:

1. Vascular underinvestment should occur in C_3_ tank-epiphytes, in association with broader leaves and low leaf mass per unit area (LMA).
2. Vascular overinvestment should occur in succulent terrestrials, in association with high extra-xylary hydraulic resistance and efficient leaf hydraulic recharge.
3. IVD:VED should be positively correlated with environmental moisture across all species, and differ accordingly between habitats.

To test these hypotheses, I characterised the relationship between IVD and VED in 376 bromeliad species representing each of the major functional types and approximately 10% of the species diversity of the entire family (Butcher and Gouda 2017). I then compared values and ratios of IVD and VED with functional type identity, bioclimatic indices, habitat data, and other key leaf traits. All three core hypotheses were supported by the data, consistent with an important role for adaptive variation in vein placement in the structure–function relationships that underpin the extraordinary ecophysiological diversity of this major radiation of tropical herbaceous angiosperms.

## Materials and methods

### Taxon sampling and leaf material

A total of 376 species were sampled in this investigation, including representatives of 7/8 subfamilies in the Bromeliaceae (full species list in Online Resource 1). Among these species were 35 C_3_ mesic terrestrials, 90 C_3_ and CAM succulent terrestrials (categorised as succulent according to morphological *Gestalt* sensu Ogburn and Edwards 2010), 150 CAM tank-epiphytes, 61 C_3_ tank-epiphytes, and 40 CAM atmospherics. Functional type assignment was made primarily on the basis of photosynthetic pathway, habit and growth-form. For 307 species, verification of photosynthetic pathway was based on the δ^13^C values reported by Crayn et al. (2015). For 17 species, δ^13^C measurements were performed on oven-dried leaf material sampled from at least three individuals using a Thermo Delta V Plus mass spectrometer (Thermo Fisher Scientific, Waltham, MA, USA) at the Godwin Laboratory, University of Cambridge. For 52 species, photosynthetic pathway was assumed on the basis of the prevailing photosynthetic pathway in the genus according to the Crayn et al. (2015) dataset. The sources of information for photosynthetic pathway verification are identified for each species in Online Resource 1, which includes all new δ^13^C values. A small proportion of species were assigned to a functional type despite displaying a different character state. For instance, a few isolated secondarily terrestrial species (e.g. *Hohenbergia catingae*) were assigned to the CAM tank-epiphytes because they are closely phylogenetically related and highly similar in morphology and physiology to the truly epiphytic species in the same group. Similarly, some putative C_3_ revertant species among terrestrial CAM genera (e.g. *Cryptanthus*) were classified as CAM terrestrials because of the uncertainty about these species’ photosynthetic flexibility and their otherwise comparable morphology and physiology. The primary habitat of each species was assigned by interrogating the eMonocot portal (http://www.e-monocot.org). Data were unavailable for two species. The following habitat categories were used: desert and xeric scrubland; Mediterranean forest and scrub; tropical dry forest; tropical moist forest; temperate mixed forest; and Andean alpine tundra.

The plants used for leaf sampling were from the living collections of four institutes: Cambridge University Botanic Garden (UK), Royal Botanic Gardens Kew (UK), Royal Botanic Gardens Edinburgh (UK), and Marie Selby Botanical Gardens (USA). All plants were grown under appropriate glasshouse or outdoor conditions, depending on the origin. The majority of species were grown in tropical glasshouses with daytime temperature of 24–30 °C, night-time temperature of 18–24 °C, and relative humidity of 85–100%. Some Chilean succulent terrestrials were grown in a subtropical glasshouse at Cambridge University Botanic Garden, with daytime temperature of 16–28 °C, night-time temperature of 12–18 °C, and RH of 50–80%. Natural and artificial illumination provided a minimal daytime photosynthetic photon flux density (PPFD) of 300 µmol m^−2^ s^−1^, which is known to be above saturating for at least 50 of the species used here when grown under the same conditions (Males and Griffiths 2017a). For outdoor-grown species (Cambridge University Botanic Garden and Marie Selby Botanical Gardens only), historical climate data are available online for Cambridge University Botanic Garden from http://www.botanic.cam.ac.uk/Botanic/Page.aspx?p=27&ix=2830 and for Marie Selby Botanical Gardens from http://www.usclimatedata.com/climate/sarasota/florida/united-states/usfl1072. Because many bromeliads are rare in cultivation, there was necessarily strong phylogenetic bias in the species sampled from different gardens, and it was not possible to include garden as a blocking factor in subsequent data analyses. However, the potential significance of interactions between growth conditions and leaf traits is covered in the Discussion.

### Anatomical measurements

**Fig. 1:**
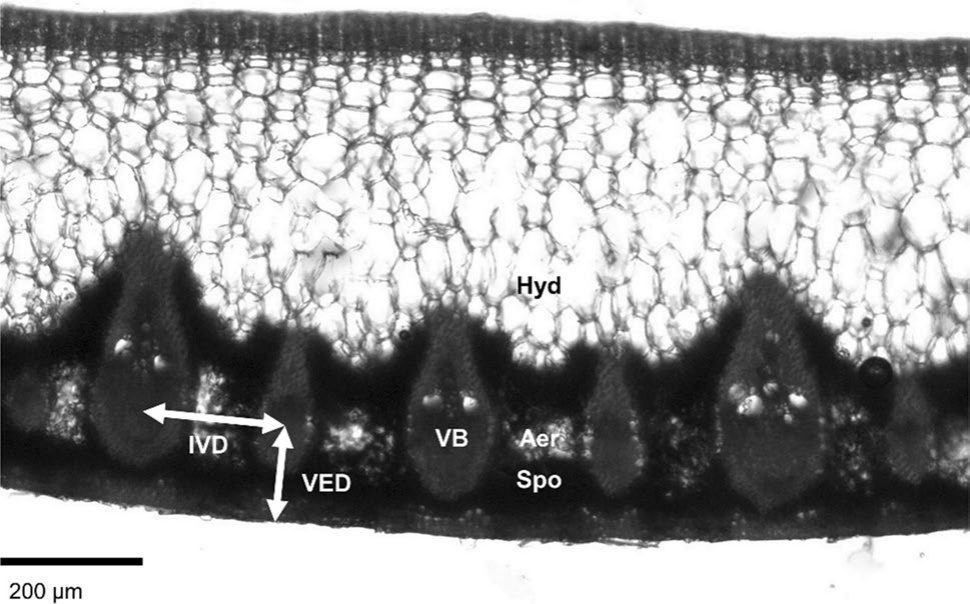
Example of transverse leaf cross-section for *Pitcairnia paniculata*, showing *IVD* interveinal distance, *VED* vein-epidermis distance, and key tissues: *Aer* aerenchyma, *Hyd* hydrenchyma, *Spo* spongy mesophyll, *VB* vascular bundle. Scale bar 200 μm

Quantification of anatomical traits was carried out according to Zwieniecki and Boyce (2014). Transverse sections were cut from the central portion of leaf blades and mounted under a light microscope (example shown in Fig. 1). Interveinal distance (IVD) was measured as the length of the straight line from the centre of one vascular bundle to the centre of an adjacent vascular bundle. Vein-epidermis distance (VED) was measured as the length of the straight line from the largest xylem conduit in a vascular bundle to the stomatiferous abaxial epidermis. All species were hypostomatous except for the amphistomatous *Catopsis berteroniana*, in which the adaxial and abaxial epidermises were equidistant from the vascular plane, meaning that the same measurement protocol could be used for VED. For succulent terrestrials, an anatomical estimate of extra-xylary apoplastic hydraulic path length was measured to test the hypothesis that this would be strongly affected by VED. This was achieved using ImageJ (NIH, Bethesda, MD, USA) to make freehand traces of the shortest apoplastic route between vascular bundles and the nearest stomata on microphotographs of the same transverse sections as used for IVD and VED measurements. Although this two-dimensional estimate cannot capture the full complexity of the extra-xylary apoplastic pathway, it offers a good approximation (Brodribb et al. 2010; North et al. 2013). All internal anatomical measurements were performed on between five and 20 leaves per species, sampled from at least three individuals depending on the living material availability.

To test the hypothesis that IVD and VED are related to leaf-blade width, leaf-blade length and their ratio in C_3_ tank-epiphytes, these traits were also measured for all species of this functional type, using the same leaves as for internal anatomical measurements.

### Leaf mass per unit area

To test the hypothesis that VED would be positively correlated with leaf mass per unit area (LMA, g m^−2^) in C_3_ tank-epiphytes, LMA values for all species of this functional type were calculated by drying and weighing leaf discs of known size sampled from the central portion of the leaf-blade. At least ten replicate discs were taken from each of the five leaves per species, sampling from at least three individuals.

### Rehydration rates

An approximate measure of rehydration rate was calculated for succulent terrestrial species to test the hypothesis that this would be affected by IVD. Initially fully hydrated leaves were allowed to dehydrate on the bench until they had lost 5% relative water content, determined by change in mass per unit leaf area and comparison with preliminary measurements of saturated leaf water per unit leaf area. Leaves were then re-cut underwater to minimise interference from embolisms, attached securely to PTFE tubing of appropriate species-specific diameter, and allowed to rehydrate through the leaf base with degassed 15 mM KCl solution. Leaves were periodically reweighed, and the time taken to return to within 1% of the initial fully hydrated mass (*t*_rehyd_) was recorded for each leaf, correcting for the tissue removed during re-cutting. Four replicate leaves were used per species, sampled from at least three individuals.

### Heuristic model of leaf hydraulic recharge

To provide an illustration of the relevance of vein placement for the process of leaf hydraulic recharge, a simple heuristic model was constructed which predicted the approximate time for full recharge on the basis of IVD and VED. Based on the preliminary results of a survey of bromeliad leaf hydraulic traits expanding on Males and Griffiths (2017a), leaf hydraulic capacitance was assumed to be logarithmically correlated with VED and leaf xylem hydraulic conductance was assumed to vary according to an inverse-log relationship with IVD. The time to full hydration (*t*_full_) was calculated for various combinations of IVD and VED as the quotient of hydraulic capacitance and xylem hydraulic conductance. While this basic approach assumes invariant tissue hydraulic conductances during recharge and does not take into account interactions with leaf morphology or transpiration, which contributes to the driving force for recharge, it does highlight the potential for vein placement to strongly affect the efficiency of leaf hydraulic recharge.

The model was developed using the following system of equations:

1a $$K_{\text{x}} = - 0.0004 \times \ln \left( {\text{IVD}} \right) + 0.0034$$

1b $$K_{\text{x}} = - 0.0004 \times \ln \left( {\text{IVD}} \right) + 0.0034$$

1c $$t_{\text{full}} = \frac{{C_{\text{FT}} }}{{K_{\text{x}} }}$$

where *K*_x_ is the leaf xylem hydraulic conductance (mol m^−2^ s^−1^ MPa^−1^), *C*_FT_ is the leaf hydraulic capacitance at full turgor (mol m^−2^ MPa^−1^), and *t*_full_ is the time (s) to full recharge. The calculation of *t*_full_ assumes the leaf is initially an empty capacitor, alongside the other assumptions outlined in the main text. While this approach is, therefore, physiologically simplistic, it captures the essential contribution of vein placement to leaf hydraulic function, and in future may be subjected to refinement when further details regarding the anatomical and biochemical determinants of bromeliad leaf hydraulic design are brought to light.

### Bioclimatic data

Bioclimatic raster datasets cannot reflect the microclimatic complexity so important to the diverse ecophysiological strategies of the bromeliads, but can nevertheless provide a very general impression of the environments in which species occur. Distributional data for all species were downloaded from the Global Biodiversity Information Facility (GBIF; http://www.gbif.org). Unreliable and non-georeferenced data were discarded, and sufficient reliable data were retained for 307/376 species. These data were passed to a script in R (R Development Core Team 2008), which retrieved mean annual precipitation (MAP, mm) and precipitation seasonality (*P*_seas_; coefficient of variation in monthly rainfall, %) values from the Bioclim database (Hijmans et al. 2005) and aridity index (AI; MAP/potential evapotranspiration, unitless) values from the Consortium for Spatial Information (CGIAR-CSI) AI database (Zomer et al. 2007, 2008) corresponding to the 30 arc-second grid-cell in which each presence point was located. Mean values of MAP, *P*_seas_ and AI were then calculated for each species. These bioclimatic variables were selected on the basis of the expectation that moisture-related environmental factors would be strong determinants of selection on IVD:VED.

### Statistical analyses

All statistical analyses were performed in R (R Development Core Team 2008). Log-transformations were performed on raw data as indicated in the text, while all figures display raw data.

## Results

**Fig. 2:**
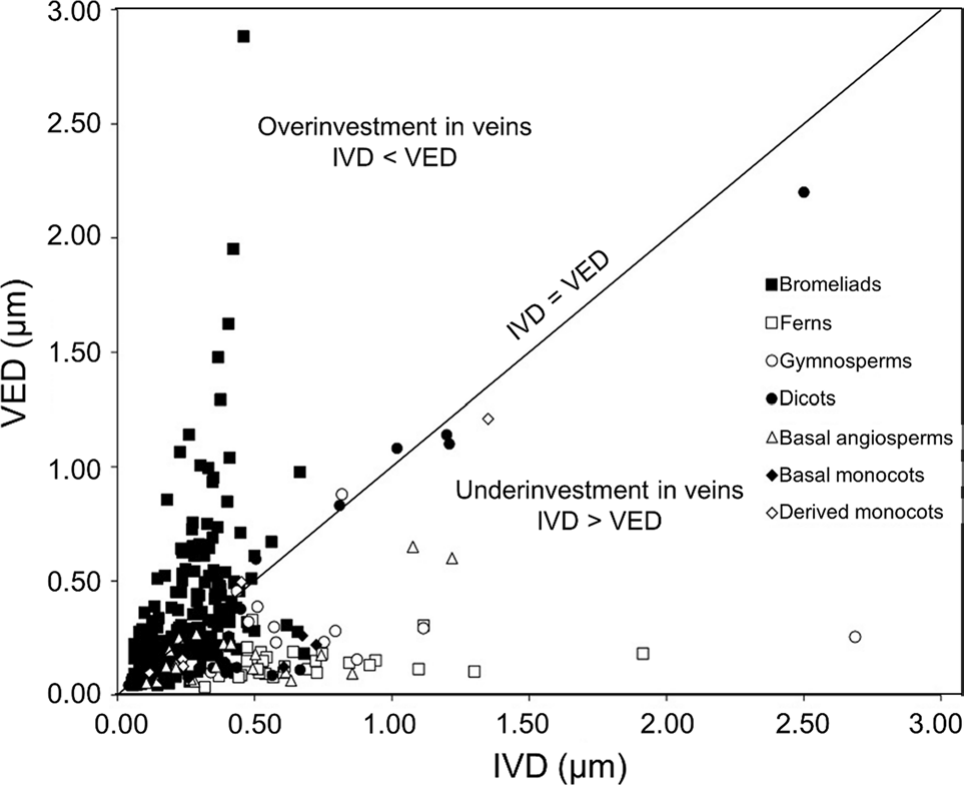
Location of 376 bromeliad species in the morphospace defined by *IVD* interveinal distance (mm) and *VED* vein-epidermis distance (mm), shown with data for other plant groups compiled by Zwieniecki and Boyce (2014). Solid line shows IVD = VED, separating morphospace regions associated with underinvestment or overinvestment in veins

Comparison of the ratios of interveinal distance (IVD) to vein-epidermis distance (VED) for the 376 bromeliad species measured in this investigation with the values reported for other plant groups by Zwieniecki and Boyce (2014) reveals that the bromeliads occupy an extremely broad swathe of IVD:VED morphospace (Fig. 2). The full dataset is available in Online Resource 1. Absolute values of IVD and VED were generally comparable with other angiosperms, although VED reached the upper limit of the values reported by Zwieniecki and Boyce (2014). Log-transformed values IVD and VED were positively correlated across all species (*r* ^2^ = 0.35, *p* < 0.001). While many bromeliad species clustered roughly along the IVD = VED line like most other angiosperms, a considerable proportion was placed far into the regions of the morphospace associated with either underinvestment or overinvestment in veins. There was a near 30-fold variation in IVD:VED, from 0.16 in the succulent Crassulacean acid metabolism (CAM) terrestrial *Hechtia purpusii* to 4.50 in the unusual petiolate succulent CAM terrestrial *Bromelia scarlatina.*

**Fig. 3:**
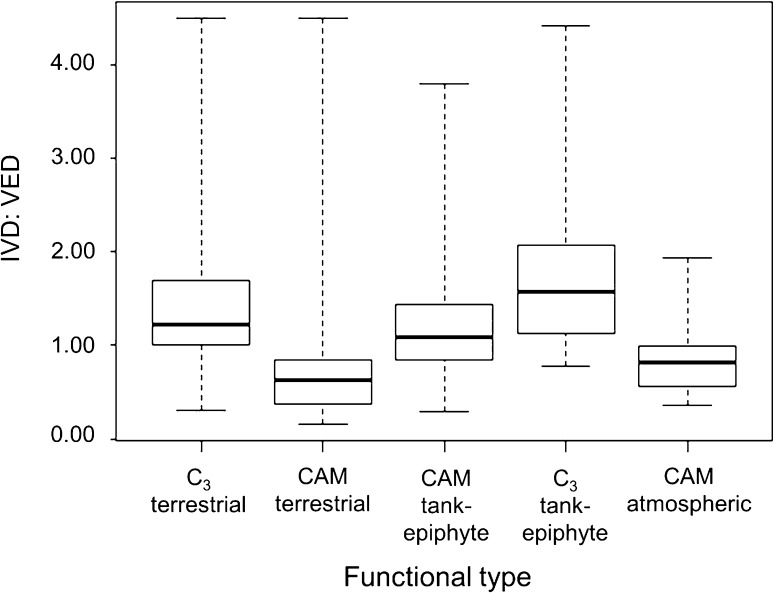
Variation in IVD:VED by functional type in the Bromeliaceae (*n* = 376)

One-way ANOVA showed that IVD:VED varied significantly between functional types (*F* = 19.89, *p* < 0.001; Fig. 3). The mean value of IVD:VED was greater than 1 in C_3_ mesic terrestrials (1.44 ± 0.13) and CAM tank-epiphytes (1.15 ± 0.04), but particularly high in C_3_ tank-epiphytes (1.71 ± 0.08), consistent with Hypothesis 1. Meanwhile, the mean value of IVD:VED was below 1 in succulent terrestrials (0.87 ± 0.08) and CAM atmospherics (0.84 ± 0.06), suggesting the prevalence of vascular overinvestment in these functional types and in accordance with Hypothesis 2.

The range of mean IVD:VED values within functional types was high in all cases, and was associated with differences in species’ morphology and ecology. Among C_3_ mesic terrestrials, an exceptionally low value occurred in the borderline succulent Guiana Shield endemic *Navia arida* (0.30). Meanwhile, the highest values occurred in mesophytic *Pitcairnia* species. Notably, the highest value (4.50) was recorded in a petiolate species with very broad leaf blades, *Pitcairnia undulata*. In the succulent terrestrials, the lowest value of IVD:VED (0.16) occurred in *Hechtia purpusii*, a strongly xeromorphic succulent. The highest value again occurred in a petiolate species with broad leaf blades, *Bromelia scarlatina* (4.50), which is native to moist Amazonian forests. Very low IVD:VED values occurred in some of the more xeromorphic CAM tank-epiphytes, including *Hohenbergia catingae* (0.29), a secondarily terrestrial species of the dry Brazilian Caatinga. Meanwhile, the highest values were measured in thin-leaved species such as the Venezuelan cloud forest endemic *Aechmea filicaulis* (3.80). The lowest IVD:VED values in the C_3_ tank-epiphytes were confined to a few large epiphytic or epilithic species such as *Alcantarea simplicisticha* (0.78). The highest value of IVD:VED occurred in the understorey/lower-canopy species *Catopsis floribunda* (4.42). Finally, among the CAM atmospherics the lowest value occurred in *Tillandsia tenuifolia* (0.36), while the highest occurred in *T. stricta* (1.94). Both these species are geographically and climatically widespread.

### Interactions with leaf morphology and LMA in C_3_ tank-epiphytes

**Fig. 4:**
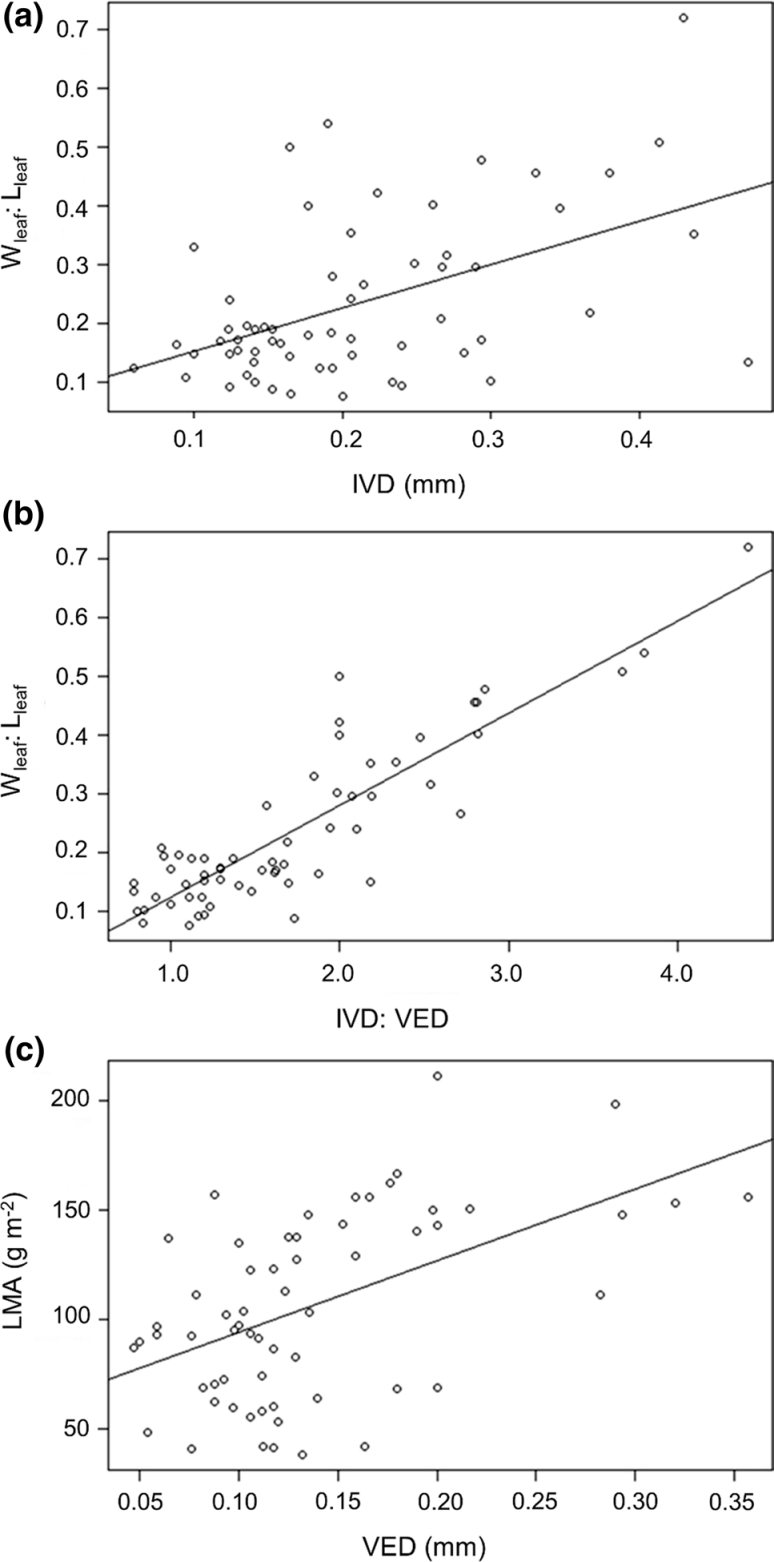
**a** Relationship between interveinal distance (IVD, mm) and ratio of leaf-blade width to leaf-blade length (*W*_leaf_:*L*_leaf_) in C_3_ tank-epiphyte bromeliads (*n* = 61). **b** Relationship between ratio of interveinal distance to vein-epidermis distance (IVD:VED) and ratio of leaf width to leaf length (*W*_leaf_:*L*_leaf_) in C_3_ tank-epiphyte bromeliads (*n* = 61). **c** Relationship between vein-epidermis distance (VED, mm) and leaf mass per unit area (LMA, g m^−2^) in C_3_ tank-epiphyte bromeliads (*n* = 61)

**Fig. 5:**
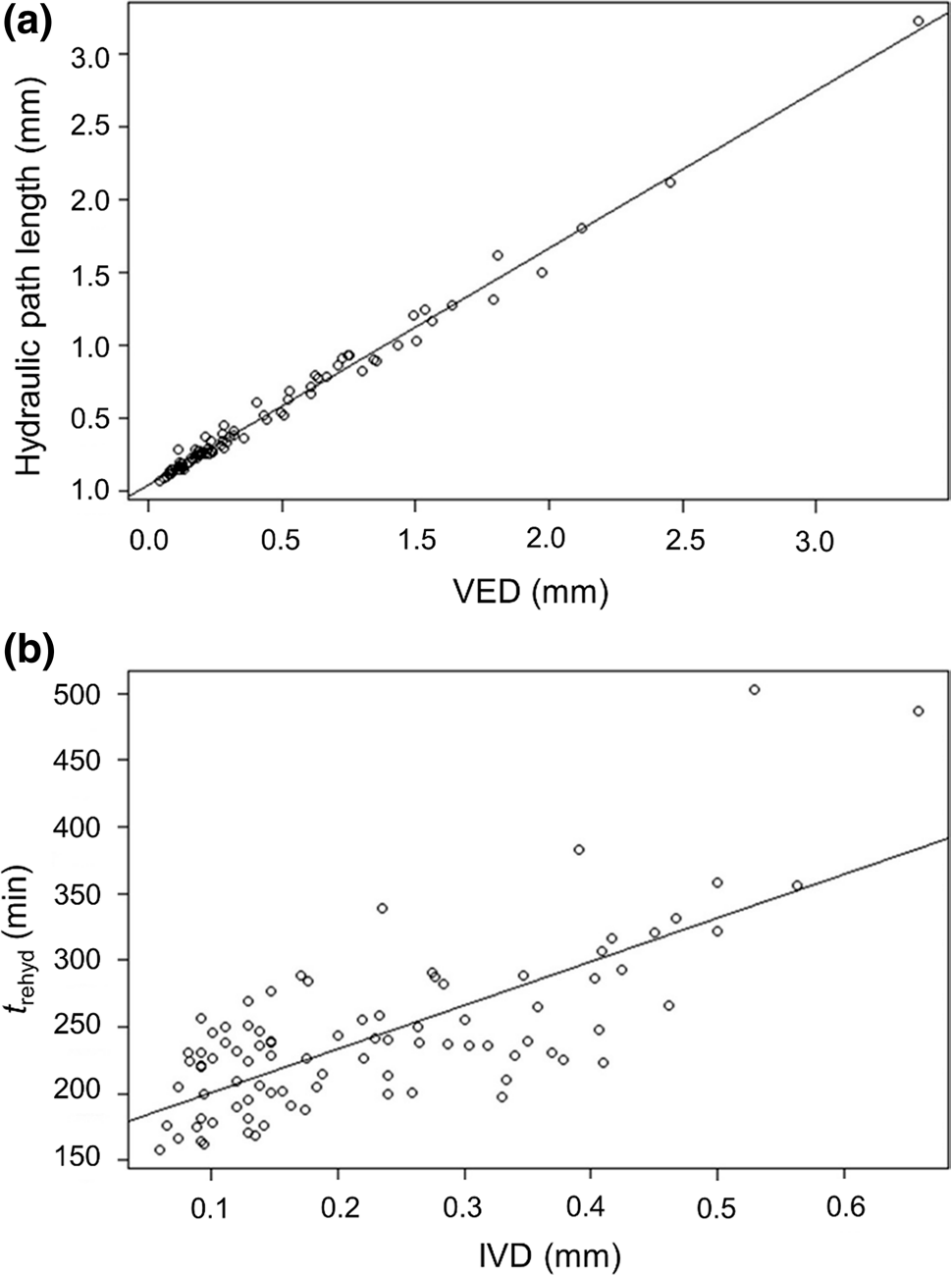
**a** Relationship between vein-epidermis distance (VED, mm) and extra-xylary apoplastic hydraulic path length (mm) in succulent terrestrial bromeliads (*n* = 90). **b** Relationship between interveinal distance (IVD, mm) and time for rehydration from 95 to 99% relative water content (*t*_rehyd_, min) in succulent terrestrial bromeliads (*n* = 90)

In the C_3_ tank-epiphytes (*n* = 61), there was a weak positive relationship between the ratio of leaf-blade width to leaf length (*W*_leaf_:*L*_leaf_) and IVD (*r* ^2^ = 0.24, *p* < 0.001; Fig. 5a), suggesting that veins tend to be more widely spaced in shorter, broader leaf blades. A stronger positive relationship was identified between *W*_leaf_:*L*_leaf_ and IVD:VED (*r* ^2^ = 0.77, *p* < 0.001; Fig. 4b), because high values of *W*_leaf_:*L*_leaf_ only occurred in species with low VED. Leaf mass per unit area (LMA) was positively correlated with VED among the C_3_ tank-epiphytes (*r* ^2^ = 0.26, *p* < 0.001; Fig. 4c), suggesting that minimisation of VED reduces the area-specific biomass investment in leaf tissue, although there was a considerable amount of unexplained variance. These results are in keeping with Hypothesis 1. Meanwhile, there was no significant correlation between LMA and *W*_leaf_:*L*_leaf_ (*r* ^2^ = −0.02, *p* = 0.892).

### Interactions with hydraulic resistance and recharge in succulent terrestrials

**Fig. 6:**
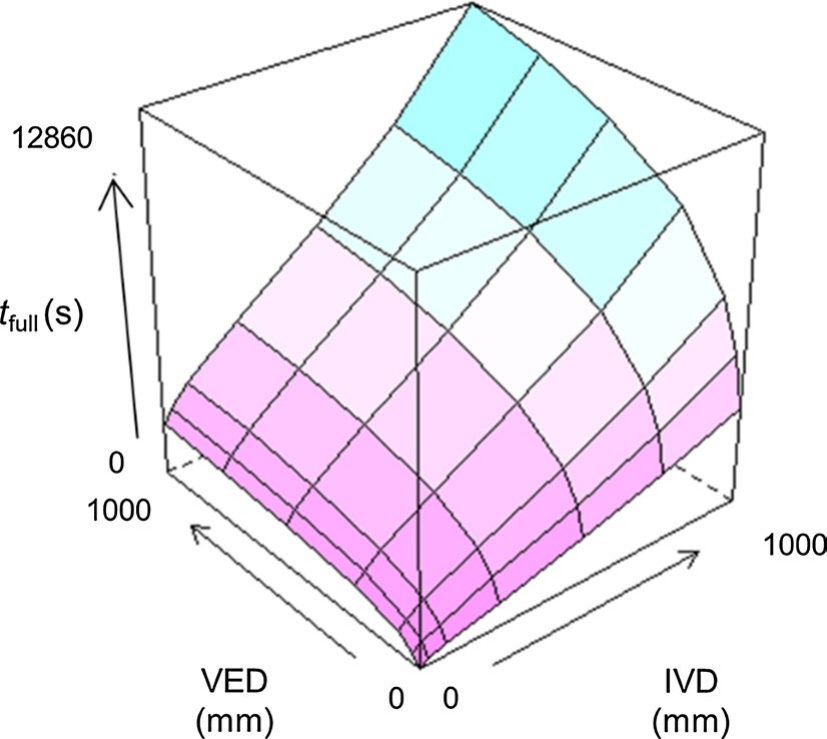
Time to full recharge (*t*_full_) in leaves with various combinations of IVD and VED, as predicted by a simple heuristic model. Low values of *t*_full_ indicate highly efficient hydraulic recharge. This figure will appear in colour online

Among the succulent terrestrials (*n* = 90), there was a strong positive linear correlation between anatomically estimated extra-xylary apoplastic hydraulic path length and VED (*r* ^2^ = 0.99, *p* < 0.001; Fig. 5a). Meanwhile, *t*_rehyd_ for succulent terrestrials varied between 158 min (*Puya laxa*) and 503 min (*Bromelia scarlatina*), and was strongly positively correlated with IVD (*r* ^2^ = 0.54, *p* < 0.001; Fig. 5b). Species with denser venation, therefore, rehydrated more rapidly, as proposed in Hypothesis 2. This contention was supported by the output of the simple heuristic model of recharge efficiency, which showed that when high VED is selected for in arid environments (because of the association between VED and hydraulic capacitance), reduced IVD tends to minimise the length of time required for full hydraulic recharge (Fig. 6). There were also weak positive correlations between log-transformed *t*_rehyd_ and extra-xylary apoplastic hydraulic path length (*r* ^2^ = 0.06, *p* = 0.012) and VED (*r* ^2^ = 0.05, *p* = 0.017), suggesting that extra-xylary structure may play a secondary role in determining hydraulic recharge efficiency.

### Bioclimatic relations and habitat affinities

**Fig. 7:**
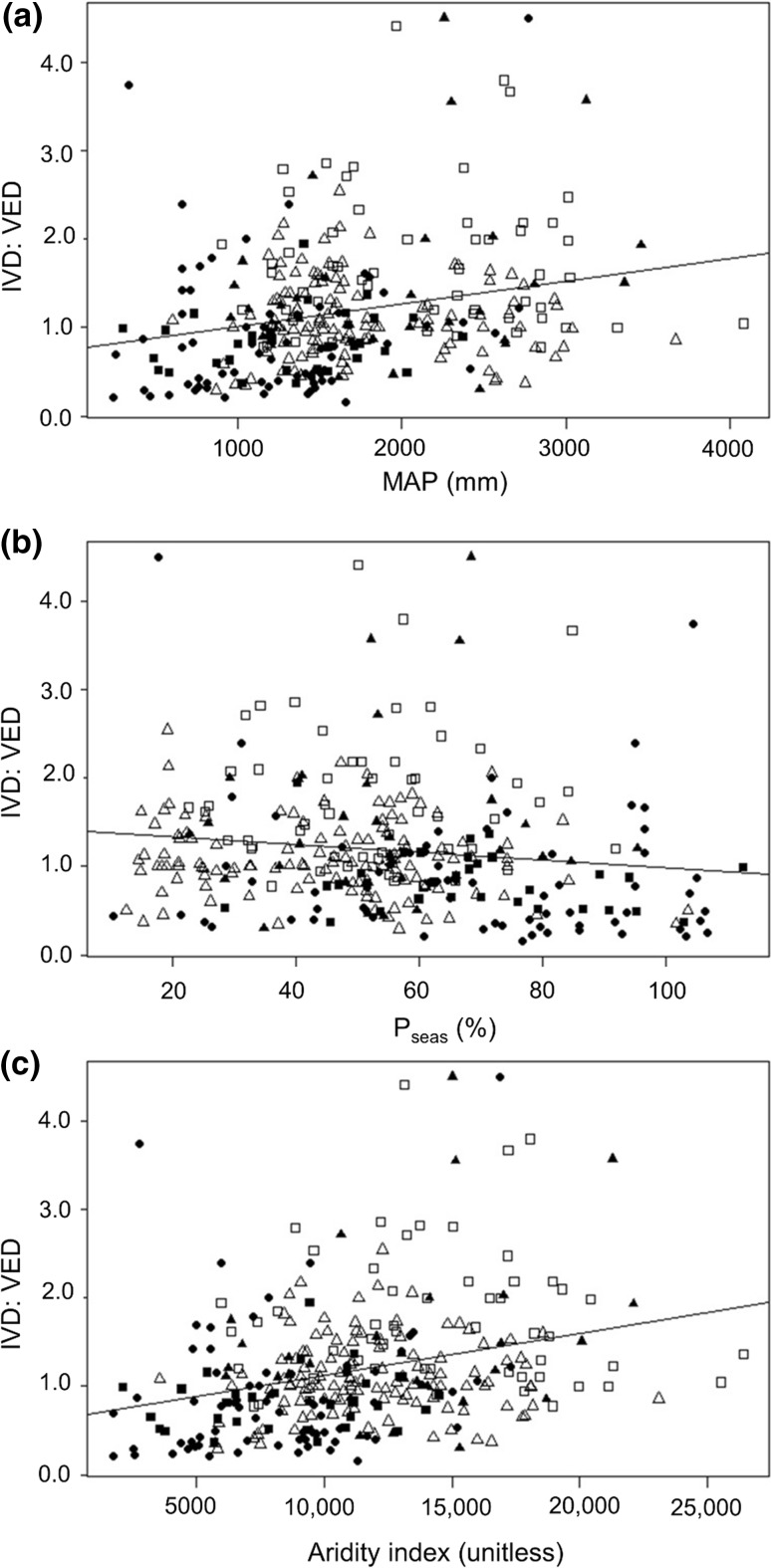
IVD:VED as a function of mean score on bioclimatic indices, with species plotted by functional type. **a** mean annual precipitation (MAP, mm); **b** precipitation seasonality (*P*_seas_, %); **c** aridity index (AI, unitless). Key to functional types: open squares—C_3_ mesic terrestrials; closed squares—succulent terrestrials; open triangles—CAM tank-epiphytes; closed triangles—C_3_ tank-epiphytes; closed circles—CAM atmospherics. Solid lines show linear regressions

Across all 307 species for which sufficient bioclimatic data were available, there were weak but statistically significant correlations between log-transformed IVD:VED and species’ mean scores for bioclimatic indices (Fig. 7). This was true for mean annual precipitation (MAP; +^ve^, *r* ^2^ = 0.09, *p* < 0.001), precipitation seasonality (*P*_seas_; −^ve^, *r* ^2^ = 0.02, *p* = 0.003), and the aridity index (AI; +^ve^, *r* ^2^ = 0.13, *p* < 0.001). The weakness of these relationships was perhaps due in part to the dominance of bromeliad ecophysiology (particularly among epiphytic species) by very fine-scale microclimatic variation that cannot be captured in global bioclimatic datasets. IVD:VED differed significantly between species associated with different habitats (*F* = 7.36, *p* < 0.001). The highest mean values of IVD:VED occurred among species from tropical moist forest (1.28 ± 0.04, *n* = 280), followed closely by temperate mixed forest (1.15 ± 0, *n* = 1) and Andean alpine tundra (1.14 ± 0, *n* = 1). A slightly lower mean value of IVD:VED occurred in Mediterranean forest and scrubland species (1.08 ± 0.14, *n* = 32), while the lowest values occurred in species from tropical dry forest (0.71 ± 0.09, *n* = 56) and desert and xeric scrubland (0.67 ± 0.33, *n* = 4).

Among the C_3_ tank-epiphytes (*n* = 61), there were no significant relationships between *W*_leaf_:*L*_leaf_ and MAP (*r* ^2^ = −0.02, *p* = 0.620), *P*_seas_ (*r* ^2^ = −0.01, *p* = 0.619), or AI (*r* ^2^ = −0.01, *p* = 0.659), nor between LMA and MAP (*r* ^2^ = −0.02, *p* = 0.697), *P*_seas_ (*r* ^2^ = 0.01, *p* = 0.193), or AI (*r* ^2^ = −0.02, *p* = 0.771). For the 69 species for which bioclimatic and rehydration data were available, there were weak positive correlations between log-transformed *t*_rehyd_ and MAP (*r* ^2^ = 0.08, *p* = 0.010) and AI (*r* ^2^ = 0.07, *p* = 0.017), but no correlation between *t*_rehyd_ and *P*_seas_ (*r* ^2^ = 0.03, *p* = 0.098).

## Discussion

The high degree of variation in the ratio of the interveinal distance (IVD) to vein-epidermis distance (VED) in the bromeliads is reflective of this plant family’s remarkable ecological diversity (Benzing 2000). The identification of significant differences in IVD:VED between functional types adds to the growing corpus of evidence linking leaf structural properties, physiological functions and ecological divergences in the bromeliads (Martin 1994; Benzing 2000; Males and Griffiths 2017a). The most striking observation, albeit in keeping with the hypotheses, was the presence of numerous cases of both marked vascular over- and underinvestment in different bromeliad lineages, which can be clearly linked to adaptive variation in other structural and functional traits.

### C_3_ tank-epiphytes: vascular underinvestment, leaf shape and LMA

In support of Hypothesis 1, vascular underinvestment was particularly strong in C_3_ tank-epiphytes from the understoreys and lower canopies of tropical moist forests. These habitats are characterised by consistently high humidity and reduced incident light, which limits the potential for vascular underinvestment to lead to hydraulic dysfunction (Zwieniecki and Boyce 2014). One important situation in which the leaf micro-environment can change very suddenly and strongly is when a sun-fleck hits the leaf. Sun-flecks are far from being fatal to understorey tank-bromeliads; they probably represent an important contribution to their carbon economy (Benzing 2000), as is the case for other understorey epiphytes (Chazdon 1988; Pearcy 1990; Chazdon and Pearcy 1991). Nevertheless, sun-flecks do pose an immediate physiological challenge, since they involve an increase not just in light intensity but also in temperature and leaf-air vapour pressure deficit (Tinoco-Ojanguren and Pearcy 1993; Schultz and Matthews 1997). This could lead to excessive water loss and destabilisation of the distribution of water potential across the lamina in species showing vascular underinvestment. To avoid hydraulic dysfunction, understorey tank-epiphyte bromeliads might be expected to rely on rapid and strong stomatal responses to environmental perturbation. Indeed, perhaps through innovation in stomatal morphology, C_3_ tank-epiphytes display more pronounced stomatal sensitivity to vapour pressure deficit and faster stomatal kinetics than C_3_ terrestrial mesophytes (Males and Griffiths 2017b).

Vascular underinvestment, and the associated thinness of the leaves, could confer several advantages on understorey epiphytic bromeliads. First, reduced vein density in the leaves could limit leaf hydraulic conductance, which, in combination with conservative stomatal behaviour, will help plants retain water in epiphytic microhabitats where this resource is often limiting (Males 2016). Secondly, as has been shown here in support of Hypothesis 1, vascular underinvestment is closely associated with increased leaf-blade width relative to length, which could be important in maximising the biomechanically supportable canopy surface area available for intercepting precipitation for refilling the plant’s central or axillary tanks (Zotz and Thomas 1999). Broader, thinner leaves will also be more efficient at exploiting diffuse light and sun-flecks in shaded forest environments (Bragg and Westoby 2002). Light limitation likely explains the occurrence in C_3_ tank-epiphyte species of a range of other visually striking adaptations, including unusual pigmentation patterns (notably pigment-based fenestration in *Vriesea* spp.; Benzing 2000) and abaxial ‘red reflectors’ (Woolley 1971). By allowing larger leaf areas to be obtained for a given nitrogen investment, pigmentation could also be indirectly linked with leaf hydraulics. Finally, the construction costs of thinner leaves are lower, enabling the production of a larger canopy for the same carbon investment. This could be of vital importance for maximising lifetime integrated water-use efficiency in epiphytic species, and may not necessarily be constrained by a trade-off with leaf longevity (Williams et al. 1989). Further work is required to elucidate the nature of the relationships between leaf structure, composition, growth, and longevity in the bromeliads.

Interestingly, some of the strongest expressions of vascular underinvestment occurred in species of the genus *Catopsis*, including exposure-demanding species that typically inhabit the crowns of emergent trees and are subjected to both high light intensities and drying winds (Benzing 2000). The advantage of vascular underinvestment in this scenario could relate to reduced leaf hydraulic conductance, perhaps in combination with very strong stomatal sensitivity. Although this would come at the price of reduced productivity, it would allow these plants to occupy an extreme epiphytic niche that is virtually free of interspecific competition.

One important focus for future research will be to disentangle the interplay between vein placement, leaf hydraulics and the longitudinal air channels or aerenchyma that are frequently observed in bromeliad leaves and are especially well developed among C_3_ tank-epiphytes (Benzing 2000; Males 2016). It is conceivable that the presence of these air channels, which might act as low-resistance conduits for water vapour as an alternative to apoplastic or symplastic water transport (Males 2016), could be the innovation that has allowed vascular underinvestment to evolve in these plants. In this connection, the presence of the air channels could have several important effects. Two key examples are that it could (1) reduce the extra-xylary apoplastic and symplastic hydraulic path lengths between the vascular bundle and the site of evaporation; and (2) facilitate rapid hydraulic equilibration between relatively distant regions of the photosynthetic mesophyll (Rockwell et al. 2014; Buckley 2015; Buckley et al. 2017). More empirical and theoretical work is needed to clarify the contribution of these enigmatic channels to bromeliad leaf physiology (Males 2016).

### Succulent terrestrials: hydraulic resistance and recharge

The succulent terrestrial bromeliads occupy a wide range of habitats and climate space (Benzing 2000). However, most occur in water-limited environments and share the capacity to take up and retain relatively large volumes of water in specialised foliar storage tissues. In these plants, much of the leaf thickness is taken up by the water storage tissue but, in the residual photosynthetic portion of the mesophyll, the vascular plane is generally set very deep relative to the abaxial epidermis. As proposed in Hypothesis 2, this results in a high extra-xylary apoplastic hydraulic path length (Brodribb et al. 2010), which is likely to make a major contribution to overall extra-xylary hydraulic resistance (Buckley et al. 2015). Further work is underway to quantify these resistances. Although symplastic hydraulic path length was not calculated here, extremely dense cell packing in the succulent terrestrial bromeliads means that it will not differ substantially from either the apoplastic path length or VED. In these species, high hydraulic resistance tends to be coupled with low stomatal density (Males and Griffiths 2017a), and the developmentally coordinated or concerted evolution of vascular and stomatal patterning in bromeliad leaves warrants further investigation.

In arid environments and on loosely structured soils, even following a moderate seasonal rainfall event, water availability can be fleeting. Succulent species occurring in such environments are likely to experience selection for rapid recharge of leaf water storage tissue to meet transpirational, metabolic and turgor requirements during the ensuing drought period (Griffiths 2013). The data presented here show that low IVD:VED can facilitate efficient hydraulic recharge, providing further support for Hypothesis 2. This observation may be generalisable to other groups of succulent plants, and merits further investigation. In the bromeliad context, this should focus on the interactions between IVD, xylem anatomy, leaf hydraulic capacitance, and the understudied functional root biology of bromeliads (Males 2016). The fact that succulent xerophytism has evolved convergently in multiple bromeliad lineages (the Xeric Clade of Pitcairnioideae, *Hechtia*, early diverging Bromelioideae with *Puya*) provides natural evolutionary replication for testing the degree of similarity in the anatomical and physiological adaptations leading to independent origins of the same ecological syndrome. Additional morphological modulation of the efficiency of recharge might be associated with leaf shape and its influence on boundary layer resistance to transpiration, which is an area with many open research questions (Males 2016).

It is worth noting that vascular overinvestment was also common in the CAM atmospheric *Tillandsia* species. These plants are entirely dependent on pulses of wetting, often from occult precipitation, for the recharge of their succulent water storage tissue (Reyes-García et al. 2008, 2012). While in theory dense venation could improve rehydration rates in these species as for the succulent terrestrials, the relative contribution of vascular water fluxes compared with absorptive trichome-mediated symplastic fluxes to the process of hydraulic recharge in these species remains poorly understood. It is conceivable that the imbalance between IVD and VED in many of these species may represent a ‘side-effect’ of developmental neoteny, a phenomenon that requires further investigation in the context of bromeliad evolutionary ecology (Benzing 2000).

### Bioclimatic relations and habitat affinities

The association of vascular overinvestment with more arid habitat types (e.g. desert and xeric scrub) and of vascular underinvestment with wetter habitats (e.g. tropical moist forest) is consistent with Hypothesis 3, and with vein placement acting as an important mediator of plant-environment interactions. Even with the relatively coarse resolution provided by global climate datasets, correlations between quantitative bioclimatic indices and IVD:VED were still recovered. While many other confounding factors (both environmental and organismal) could limit the strength of these relationships, it is possible that they would appear stronger if finer-scale (regional/landscape-level) or microclimatic data were available. To address the question of which spatial scales are meaningful for trait-climate relationships, future research could focus on establishing whether leaf anatomy–physiology–climate relationships can be detected in the distributions of bromeliad species at the landscape scale, ideally making use of fine-scale locally collected climate data and using experimental work to elucidate the processes underlying the correlations.

There are still relatively few examples of investigations into trait-mediated environmental niche differentiation among herbaceous vascular plants in the Neotropics, due in part to a persistent bias towards research into woody species. The development of process-oriented, trait-based explanations for bromeliad species distributions along environmental gradients would not only improve our understanding of bromeliad evolutionary ecology, it could also inform theoretical and applied conservation research aimed at clarifying and mitigating the potential impacts of global change on vulnerable species. Additionally, when robust species-level phylogenies become available for more bromeliad genera, this will present an opportunity for performing powerful analyses of the evolution of trait-defined environmental niches in a comprehensive, phylogenetically explicit framework.

### Interactions with other aspects of leaf structure

It is intriguing to note the apparent connection between vein placement traits, environmental niches and particular leaf shapes, some of which have evolved convergently in different bromeliad lineages. The best example is provided by the petiolate morphology that has arisen independently in several bromeliad genera, and was represented in this dataset by species in *Aechmea*, *Bromelia*, *Cryptanthus*, *Disteganthus*, *Fosterella* and *Pitcairnia*. Petiolate species seem always to be restricted to very moist, usually forested environments. They appear to be characterised by high values of IVD:VED, which could represent an important structural determinant of their water-use strategies. Two other anatomical aspects of bromeliad leaf structure that could interact with IVD:VED to modulate physiological function and environmental tolerances are the structural composition of the xylem and the hierarchical architecture of the venation (McKown et al. 2010; Sack and Scoffoni 2013). While the present investigation did not include an assessment of these factors, further study of this subject is underway.

### Plasticity in leaf form and function: the unknown quantity

Very few quantitative data are currently available regarding the potential for morphological plasticity in bromeliad leaves in response to environmental variability (e.g. Scarano et al. 2002; Cavallero et al. 2009). All plant material used in this investigation was grown under glasshouse or garden conditions, and kept well watered. If the capacity for environmentally induced phenotypic plasticity in any given species is high, quite different IVD:VED values could be observed under natural conditions in the field. As has been observed in other plant groups (e.g. Scoffoni et al. 2015), this could have important implications for physiological function, environmental tolerance, and niche breadth and there is, therefore, an urgent need for more focussed studies of plasticity within bromeliad species.

## Conclusions

The results of this investigation are consistent with an important role for leaf vein placement in modulating species’ ecological niches. In the bromeliads, vein placement parameters differ significantly between functional types. Vascular underinvestment is especially common among C_3_ tank-epiphytes with broad, thin leaf blades and inhabiting moist, low-light microhabitats in tropical forests, probably in association with resource foraging. Vascular overinvestment is common to multiple lineages of succulent terrestrials, and confers the dual benefit of increased extra-xylary hydraulic resistance and increased efficiency in the refilling of water storage tissue during brief pulses of water availability. More generally, there is a trend towards vascular overinvestment in more water-limited environments, while vascular underinvestment is more frequent in high-moisture environments. Future research should take into account interactions between vein placement and other aspects of leaf anatomical structure and physiological function in the pursuit of a more complete understanding of how change in leaf traits has mediated ecological divergences and contributed to evolutionary diversification in this important plant family, as well as in other angiosperm radiations involving vegetative specialisation.

## Electronic supplementary material

Below is the link to the electronic supplementary material.

Supplementary material 1 (XLSX 70 kb)
